# Disulfiram ameliorates bone loss in ovariectomized mice by suppressing osteoclastogenesis

**DOI:** 10.1007/s00774-024-01555-x

**Published:** 2024-10-07

**Authors:** Tatsuyuki Fukui, Asuka Terashima, Yasunori Omata, Ryota Chijimatsu, Kazuo Okamoto, Masayuki Tsukasaki, Yukiko Fukuda, Tadayoshi Hayata, Akiyoshi Saitoh, Etsuko Toda, Hiroshi Takayanagi, Sakae Tanaka, Yuya Terashima, Taku Saito

**Affiliations:** 1 Sensory and Motor System Medicine, Graduate School of Medicine, The University of Tokyo, 7-3-1 Hongo, Bunkyo-ku, Tokyo 113-8655 Japan; 2 Bone and Cartilage Regenerative Medicine, The University of Tokyo Hospital, Tokyo, 7-3-1 Hongo, Bunkyo-ku, Tokyo 113-8655 Japan; 3 Center for Comprehensive Genomic Medicine, Okayama University Hospital, Shikata-cho, Kita-ku, Okayama 700-8558 Japan; 4 Department of Osteoimmunology, Graduate School of Medicine, The University of Tokyo, 7-3-1 Hongo, Bunkyo-ku, Tokyo 113-0033 Japan; 5 Division of Immune Environment Dynamics, Cancer Research Institute, Kanazawa University, Kakuma-Machi, Kanazawa, 920-1192 Japan; 6 Laboratory of Pharmacology, Graduate School of Pharmaceutical Sciences, Tokyo University of Science, 2641, Yamazaki, Noda, Chiba 278-8510 Japan; 7 Division of Molecular Regulation of Inflammatory and Immune Diseases, Research Institute for Biomedical Sciences, Tokyo University of Science, 2641, Yamazaki, Noda, Chiba 278-8510 Japan; 8 Department of Molecular Pharmacology, 2641, Yamazaki, Noda, Chiba 278-8510 Japan; 9 Department of Analytic Human Pathology, Nippon Medical School, 1-25-16, Nezu, Bunkyo-ku, Tokyo 113-0031 Japan; 10 Department of Immunology, Graduate School of Medicine and Faculty of Medicine, The University of Tokyo, 7-3-1 Hongo, Bunkyo-ku, Tokyo Japan

**Keywords:** Osteoporosis, DSF, Osteoclast precursor, Osteoclastogenesis, scRNA-seq analysis

## Abstract

**Introduction:**

Disulfiram (DSF), known as an anti-alcoholism drug, has been reported to suppress osteoclast differentiation in vitro; however, it remains uncertain whether DSF is effective in preventing osteoclastogenesis in vivo. This study aimed to investigate the effect of DSF administration in osteoporotic mice and its contribution to osteoclastogenesis in vivo.

**Materials and methods:**

The bone phenotype of ovariectomized mice, both treated and untreated with DSF, was examined using microcomputed tomography analysis. Osteoclastic and osteoblastic parameters were assessed through bone morphometric analysis. The direct effect of DSF on osteoblastogenesis in vitro was evaluated via a primary osteoblast culture experiment. The expression of genes related to DSF targets (*Nup85*, *Ccr2*, and *Ccr5*) in osteoclast-lineage cells was examined using scRNA-seq analysis and flow cytometry analysis using the bone marrow cells from ovariectomized mice. The impact of DSF on osteoclast-lineage cells was assessed using primary cultures of osteoclasts.

**Results:**

DSF administration ameliorated ovariectomy-induced bone loss and mitigated the increase of osteoclasts without affecting osteoblastogenesis. The scRNA-seq data revealed that osteoclast precursor cells expressed *Nup85*, *Ccr2*, and *Ccr5*. CCR2 and CCR5-positive cells in osteoclast precursor cells within bone marrow increased following ovariectomy, and this increase was canceled by DSF administration. Finally, we found that DSF had a significant inhibitory effect on osteoclastogenesis in the early stage by suppressing *Tnfrsf11a* expression.

**Conclusion:**

This study demonstrates that DSF could be a candidate for osteoporosis therapies because it suppresses osteoclastogenesis from an early stage in vivo.

**Supplementary Information:**

The online version contains supplementary material available at 10.1007/s00774-024-01555-x.

## Introduction

Osteoporosis is a serious health concern in the global community. Approximately, 75 million people are estimated to have osteoporosis in the United States, Europe, and Japan [1]. Osteoporosis causes bone fragility and increases the risk of fractures. Enhanced bone resorption is observed in most postmenopausal women and elderly individuals, leading to osteoporosis [2]. Currently available anti-osteoporotic drugs are mainly classified into two groups: anti-resorptive and anabolic agents [3]. Bisphosphonates and denosumab are classified as anti-resorptive agents that suppress osteoclast functions and reduce bone remodeling [4, 5]. They are widely used to mitigate bone loss associated with senescence or menopause. However, these agents are known to suppress not only bone resorption but also bone formation [6], resulting in decreased bone turnover, which can lead to complications such as osteonecrosis of the jaw [7–9] or atypical femoral fracture [10]. Therefore, alternative options for osteoporosis treatment are still being explored.

Osteoclasts are unique cells that possess bone resorption activity and play a critical role in bone metabolism, making them one of the therapeutic targets for osteoporosis. They are multinucleated large cells formed by the fusion of mononuclear monocyte–macrophage lineage progenitor cells [11]. The maturation of osteoclast progenitor cells is primarily initiated by the stimulation of two key cytokines: macrophage colony-stimulating factor (M-CSF) and receptor activator of nuclear factor-kappa B ligand (RANKL). Both cytokines are produced by osteoblasts, osteocytes, and bone marrow (BM) stromal cells [12, 13]. Following cytokine stimulation, the expression of the transcription factor nuclear factor activated T cells c1 (NFATc1), which functions as a master regulator for osteoclast differentiation [14], is induced, leading to the expression of osteoclast-specific genes such as *Acp5*, *Ctsk*, and *Mmp9* [14].

Disulfiram (DSF), initially reported as an aldehyde dehydrogenase (ALDH) inhibitor, which causes severe alcohol intolerance, has been used to treat alcoholism [15]. We reported that DSF has a suppressive effect on FROUNT, which is encoded by the *Nup85* gene [16], and that FROUNT regulates monocyte and macrophage migration by binding to C–C motif chemokine receptors (CCR) 2 and CCR5 [17, 18]. Based on these findings, we have verified and reported the potential therapeutic effects on various diseases, including cancer, anxiety, glomerulonephritis, and corneal alkali burn [16, 19–21]. Furthermore, DSF has also been reported to modulate mitogen-activated protein kinase (MAPK) and the nuclear factor-kappa B (NF-κB) pathways [22]. In addition, DSF has a suppressive effect on osteoclast differentiation in vitro [23]. The report showed that DSF suppresses ERK phosphorylation and impairs nuclear translocation of p65 (a core component of NF-κB), resulting in the inhibition of NFATc1 expression. However, this report did not show the effect of DSF on osteoclastogenesis in in vivo models.

In this study, we examined the effect of DSF administration on osteoporotic mice and analyzed how DSF affects osteoclastogenesis in vivo. Our findings reveal that DSF ameliorates osteoporotic changes induced by ovariectomy (OVX) via the suppression of osteoclast differentiation without affecting osteoblast functions. We focused on a target of OVX, FROUNT, and its binding receptors CCR2 and CCR5, in osteoclast precursor cells (OCPs). Single-cell RNA sequencing (scRNA-seq) analysis revealed the expression of CCR2 and CCR5 expression in OCPs, and these expressions are increased in the OCPs of OVX-operated mice. The increased expressions of these molecules are but was canceled by DSF administration in vivo. Furthermore, administration of DSF before RANKL stimulation in vitro significantly reduced the expression of *Tnfrsf11a*, which is the gene for the receptor of RANKL. Our study suggests that DSF could be a candidate for treating osteoporosis by suppressing osteoclastogenesis while maintaining osteoblast functions.

## Materials and methods

### Animals

C57BL/6J mice were purchased from Sankyo Lab Services (Tokyo, Japan). All animal experiments were performed in accordance with the guidelines described in the Guide for the Use and Care of Laboratory Animals of the Institute for Laboratory Animal Research (ILAR, 2011) and were approved by the Institutional Animal Care and Use Committee (IACUC) of The University of Tokyo (M-P17-091). Animals were kept under the supervision of the IACUC at 20–26 °C with a 12-h light/dark cycle and were provided unrestricted access to food and water.

### Animal models

Eight-week-old female mice were anesthetized with an intraperitoneal injection of 0.3 μg/g body weight medetomidine chloride, 4 μg/g body weight midazolam, and 5 μg/g body weight butorphanol tartrate. After confirming no signs of wakefulness, the mice were placed in a prone position. To remove the ovaries, a transverse incision was made in the back around the lumbar spine to expose the retroperitoneum. Following the removal of the ovaries, the skin was sutured. DSF (NOCBIN; Mitsubishi Tanabe Pharma Corp., Osaka, Japan) or vehicle, 2-hydroxypropyl-β-cyclodextrin (HBC; Wako, Osaka, Japan), was administered intraperitoneally at a dose of 40 mg/kg once daily for 5 days per week. Weight gain and/or visual uterine atrophy were assessed in all ovariectomized mice to confirm the successful removal of the ovaries. The fifth lumbar vertebrae were harvested from the mice and fixed with 4% paraformaldehyde (PFA) or 70% ethanol at 4 °C for microcomputed tomography analysis and histology.

### Microcomputed tomography analysis

All bone samples were scanned using the inspeXio SMX-100CT Microfocus X-Ray CT System. Subsequently, 3D bone images were constructed from the slice data. Bone volume per total volume (BV/TV), trabecular number (Tb.N), and trabecular separation (Tb.Sp) were analyzed and calculated using a TRI/3D-BON-FCS64 (RATOC System Engineering, Tokyo, Japan).

### Tartrate-resistant acid phosphatase (TRAP) staining

The harvested vertebrae were fixed with 4% (w/v) PFA at room temperature overnight, followed by decalcification with a 10% (w/v) ethylenediaminetetraacetic acid solution at 4 °C for 1 week. Following the decalcification, specimens were dehydrated, immersed, and embedded in paraffin. Tissue sections, cut at a thickness of 4 μm, were stained using a TRAP staining kit (AK04F, Cosmo Bio, Ehime, Japan). Nuclei in the tissue sections were stained with Mayer’s hematoxylin. The number of TRAP-positive cells per 1 mm of bone surface (N.Oc/BS) was quantified using NIH ImageJ software.

### Histomorphometric analysis

Histomorphometric analysis was performed on undecalcified sections of the fifth vertebral bone. For double calcein labeling, mice were subcutaneously administered with calcein at a dose of 16 mg/kg on Days 4 and 1 before killing. Toluidine blue staining was used for the analysis.

### Osteoblast differentiation from murine calvarial cells

Calvarial cells were isolated from newborn C57BL/6J mice (1–3 days old) through enzymatic digestion in α-minimum essential medium (α-MEM) with 0.1% collagenase (Wako) and 0.2% Dispase (Roche, Basel, Switzerland). The cells were cultured with α-MEM containing 10% fetal bovine serum (FBS) and 1% penicillin–streptomycin. The day after seeding the isolated cells, the culture media were changed to remove non-adherent cells. After 3 days, the cells were reseeded (1.5 × 10^4^ cells per well) and cultured with osteogenic medium (α-MEM containing 10% FBS, 1% penicillin–streptomycin, 50 μg/mL ascorbic acid (Sigma-Aldrich, St. Louis, USA), 10 mM β-glycerophosphate (Sigma-Aldrich), and 10 nM dexamethasone (Sigma-Aldrich)). Media changes occurred every 2–3 days. Osteoblast differentiation was assessed by evaluating the alkaline phosphatase (ALP) activity and mineralization. ALP activity was detected using BCIP/NBT color development substrate (Promega, Madison, WI, USA), and mineralization was assessed with 1% Alizarin Red S Solution (pH 6.4) (Muto Pure Chemicals, Tokyo, Japan). The colored area of each well was measured using ImageJ software.

### Single-cell RNA sequencing analysis

A publicly available dataset (Gene Expression Omnibus database, GSE147174) was utilized for the analysis. The analysis was performed using the Seurat R software package (version 4.2.2) and the RStudio software package (version 2022.07.2 + 576). Cells with unique feature counts over 5,000 or less than 500 and > 15% mitochondrial counts were filtered out. Read counts per cell were normalized and scaled based on a unique molecular identifier count using the ‘SCTransform’ function [24]. Genes with significantly varying expression were identified by the same ‘SCTransform’ function. Normalized expression values were subjected to principal component analysis (PCA) using the ‘RunPCA’ function for dimensionality reduction. Subsequently, using the ‘ElbowPlot’ function to determine significant principal components, we retained the first 30 principal components for UMAP projection. Clusters were identified by constructing a shared nearest-neighbor graph using the top 50 dimensions of the PCA and 20 k-nearest neighbors with the ‘FindNeighbors’ function before applying the Louvain algorithm for modularity optimization with the ‘FindClusters’ function. In addition, the clustree package was used to determine the clustering resolution [25].

### Flow cytometry and antibodies

BM cells were isolated by flushing from the murine femur. Red blood cells among BM cells were lysed using red blood cell lysis solution (pluriSelect, Leipzig, Germany). The antibodies used for the flow cytometric analysis, aiming to detect OCPs were as follows: BV 510 anti-mouse/human CD11b (M1/70), PE-Cy7 anti-mouse CD115/CSF-1R (AFS98), PE anti-mouse CD192/CCR2 (SA203G11), PerCP-Cy5.5 anti-mouse CD195/CCR5 (HM-CCR5), FITC streptavidin, biotinylated anti-mouse CD3e (145-2c11), CD45R/B220 (RA3-6B2), Ly-6G (1A8), NK-1.1 (PK136), and TER-119 (TER-119). To detect TNFRSF11A-positive cells, anti-mouse CD265 antibodies (R12-31) were used. OCPs were defined as Lin^−^ CD11b^−/lo^ CD115^+^ cells. All antibodies were purchased from BioLegend. The flow cytometric assay was performed using CytoFLEX S (Beckman-Coulter, USA), and the obtained data were analyzed using Kaluza software (Beckman-Coulter).

### Osteoclast differentiation from murine BM cells

BM cells obtained from 8- to 10-week-old C57BL/6J mice were cultured with α-MEM containing 10% FBS and human M-CSF (R&D Systems, Minneapolis, USA) for 2 days. Subsequently, these cells were cultured for an additional 3 days in media with human RANKL (Wako). The culture media were changed once every second day in all experiments. DSF was added according to the procedure outlined in Fig. 6a. Osteoclastogenesis was evaluated by manually counting the number of TRAP-positive multinucleated cells (i.e., more than three nuclei) and calculating the percentage of their occupied surface area using the imaging software BZ-X710 (Keyence Corporation, Osaka, Japan).

### Quantitative reverse transcription polymerase chain reaction (qPCR)

Total RNA was extracted using a Direct-zol RNA Kit (Zymo Research, Irvine, CA) in accordance with the manufacturer’s protocol. The total RNA was reverse-transcribed into cDNA using ReverTra Ace qPCR RT Master Mix (TOYOBO, Osaka, Japan). The qPCR was performed using THUNDERBIRD SYBR qPCR Mix (TOYOBO) and a Thermal Cycler Dice Real-Time System III (Takara Bio, Shiga, Japan). Information regarding the primers is provided in Supplementary Table 1. The ΔΔCT method was used to measure the expression levels, which were normalized to hypoxanthine phosphoribosyltransferase 1 (*Hprt1*).

### Statistical analysis

The statistical significance between groups was determined using GraphPad 9.1.1 software. Data are presented as dot plots with a line representing the mean and error bars indicating the variances among groups. The *p*-values and statistical methods are shown in each figure legend.

## Results

### DSF ameliorates the increase in osteoclast number and the osteoporotic phenotype induced by OVX

**Fig. 1:**
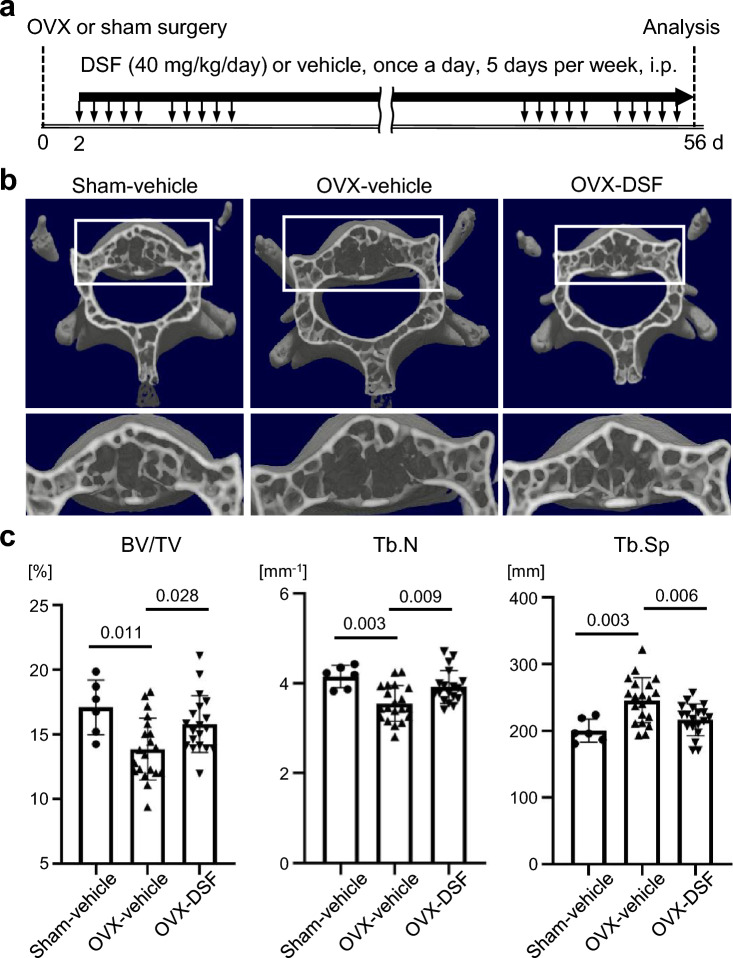
DSF administration improves the OVX-induced bone loss. **a** Schematic illustration of the time schedule for OVX and administration of DSF or vehicle. **b** Representative μCT images of the lumbar vertebrae of sham-operated mice treated with vehicle and OVX-operated mice treated with vehicle or DSF. **c** Vertebral architectures. BV/TV, bone volume per total volume; Tb.N, trabecular number; Tb.Sp, trabecular separation. Data are presented as the mean (SD), and *p-*values were determined using Tukey’s multiple comparisons test. Significant *p-*values are shown

**Fig. 2:**
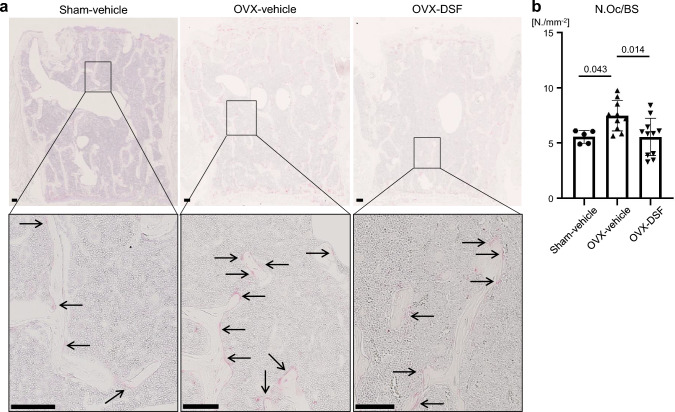
DSF administration ameliorates the increased osteoclast number in OVX mice. **a** Representative images of TRAP staining of the vertebral tissue section. Arrows indicate osteoclasts. Scale bars, 100 μm. **b** Quantitative analysis of TRAP-positive osteoclast numbers. Data are presented as the mean (SD), and *p-*values were determined using Tukey’s multiple comparisons test. Significant *p-*values are shown. N.Oc/BS, osteoclast number per bone surface

To investigate the effects of DSF on bone metabolism in an osteoporotic condition, we first examined the bone phenotype of ovariectomized mice (a model of osteoporosis) treated with DSF. DSF or vehicle was administered intraperitoneally to the ovariectomized mice at a dose of 40 mg/kg once a day for 5 days per week (Fig. 1a). We also compared sham-operated mice treated with either vehicle or DSF to determine whether DSF affects bone metabolism under healthy conditions. DSF had no effect on bone parameters under normal conditions (Supplementary Fig. 1). Bone phenotype was assessed at 8 weeks after OVX. Microcomputed tomography analysis showed that BV/TV and Tb.N were decreased, and Tb.Sp was increased in OVX mice treated with vehicle (Fig. 1b, c). In contrast, the administration of DSF to OVX mice significantly prevented the OVX-induced bone loss (Fig. 1b, c). Since OVX surgery is known to stimulate osteoclast activation and DSF has the potential to inhibit osteoclast formation [26], we measured the number of osteoclasts in our model. There was a significant difference in osteoclastic parameters between OVX mice treated with vehicle and DSF at 8 weeks after OVX surgery (Supplementary Fig. 2). In addition, to examine the osteoclast phenotype at an earlier stage, we assessed the number of osteoclasts at 1 week after surgery. We found that the osteoclast number in the OVX mice treated with vehicle was increased compared with sham mice treated with vehicle. On the other hand, the OVX-induced increase in the TRAP-positive osteoclast number was suppressed by the DSF treatment (Fig. 2a, b). These results indicate that DSF has a therapeutic effect on OVX-induced bone loss by inhibiting the formation of osteoclasts.

### DSF has no obvious effect on osteoblastogenesis either in vivo or in vitro

**Fig. 3:**
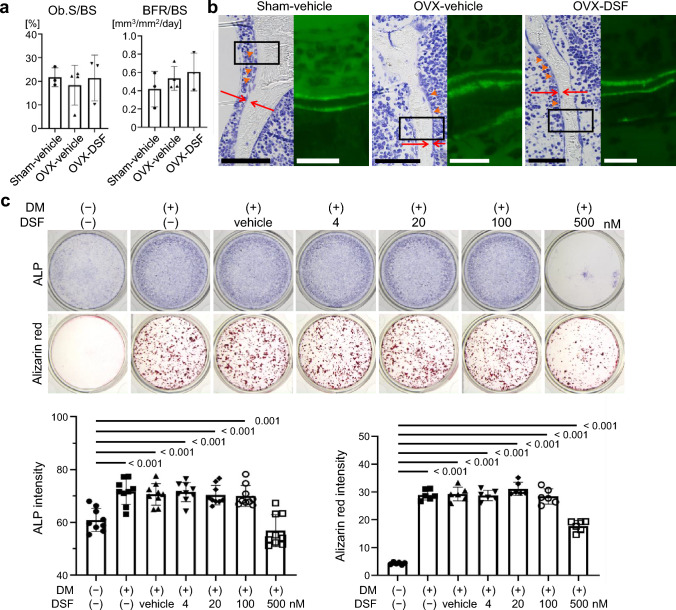
DSF administration has no obvious effect on osteoblast function and differentiation either in vivo or in vitro. **a** Histomorphometric analysis of the lumbar vertebrae. **b** Representative images in histological sections of the lumbar vertebrae of toluidine blue staining (left) and calcein double labeling for determining bone formation (right). Osteoids are indicated between red arrows, and orange arrowheads indicate osteoblasts. Black bars, 20 μm. White bars, 5 μm. **c** (upper) Representative images of alkaline phosphatase (ALP) staining and alizarin red S staining of murine cranial cells incubated in osteogenic media. (lower) Quantitative analysis of the stain intensity for ALP staining and Alizarin Red S staining. Data are presented as the mean (SD), and *p*-values were determined using Tukey’s multiple comparisons test. Significant *p*-values are shown. Ob.S/BS, osteoblast surface per bone surface; BFR/BS, bone formation rate per bone surface; DM, differentiation medium

Next, we analyzed osteoblastic parameters to examine the contribution of DSF to bone formation in our model. Bone metabolism is regulated by both osteoclastic bone resorption and osteoblastic bone formation [27, 28]. Osteoblastic parameters (Ob.S/BS and BFR/BS) were evaluated by bone morphometric analysis using the vertebrae of the mice. One week after the OVX surgery, which corresponds to the same timing as in Fig. 2a and 2b, no significant changes were observed in the osteoblastic parameters in the DSF-treated mice (Fig. 3a, b). These data suggest that DSF did not affect bone formation regarding bone mass in vivo. We then assessed the effect of DSF administration on osteoblast differentiation using murine calvarial cells in vitro (Fig. 3c). The results showed that DSF treatment had no direct effect on osteoblastogenesis at drug concentrations below the IC_50_ (214.95 ± 14.78 nM) [23]. These findings indicate that DSF does not inhibit osteoblastic bone formation or osteoblast differentiation.

### Osteoclast precursor cells express a target of DSF

**Fig. 4:**
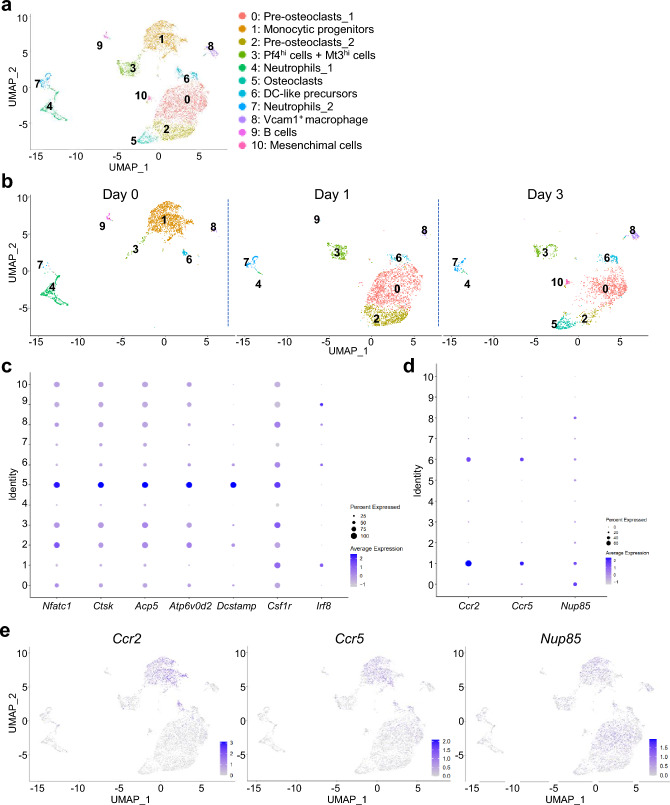
Osteoclast precursor cells (OCPs) express *Ccr2, Ccr5*, and *FROUNT* based on scRNA-seq analysis. **a** Uniform manifold approximation and projection (UMAP) visualization of 11 clusters in the osteoclast culture system. **b** Separated UMAPs at Days 0, 1, and 3. **c** Dot plot showing the expression of osteoclast marker genes in the identified clusters. **d** Dot plot showing the expression of *Ccr2, Ccr5,* and *FROUNT* in the identified clusters. **e** Expression of *Ccr2, Ccr5,* and *FROUNT* genes in the UMAP visualization

**Fig. 5:**
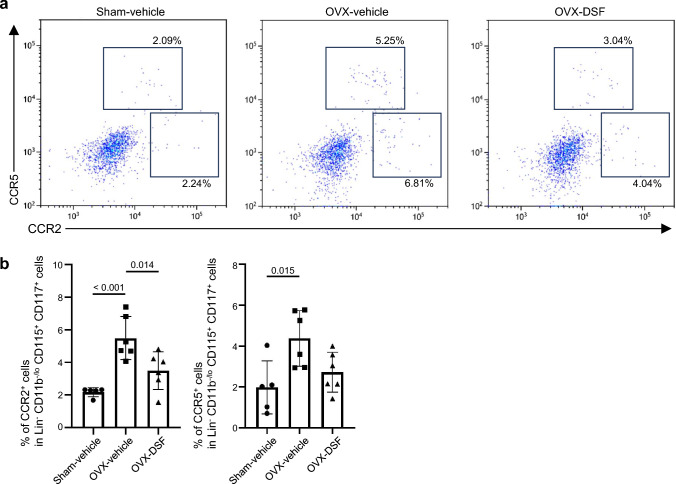
DSF administration suppresses the increase in the percentages of CCR2- or CCR5- positive cells in the OCPs in OVX mice. **a, b** Percentage of CCR2- (**a**, lower right box) or CCR5- positive cells (**a**, upper box) in Lin^−^ CD11b^−/lo^ CD115^+^ CD117^+^ OCPs. The data are representative of more than two independent experiments. Data are presented as the mean (SD), and *p-*values were determined using Tukey’s multiple comparisons test. Significant *p-*values are shown

To ascertain the potential effect of DSF on osteoclast-lineage cells, we tried to examine the gene expression profiles of *Nup85*, *Ccr2*, and *Ccr5* in them. For the analysis, we used a publicly available data set (Gene Expression Omnibus database, GSE147174). The “day 0” scRNA-seq data in Fig. 4b were obtained from mouse BM cells cultured with M-CSF for 2 days. Subsequently, “Day 1” and “Day 3” data were obtained from cells cultured for one and three additional days with RANKL in the presence of M-CSF, respectively. Cluster 1 was the major component of cells harvested on Day 0, suggesting that this cluster contained OCPs. In addition, this cluster expressed mainly M-CSF receptor (*Csf1r*) and a part of the cluster expressed *Irf8*, which is a negative regulator for osteoclastogenesis [29]. Therefore, the cluster should be composed of monocytic progenitors (Fig. 4a–c) including OCPs. Since clusters 0 and 2 were the main components of cells stimulated by RANKL for 24 h (Day 1), they were presumed to contain preosteoclasts (Fig. 4a). Indeed, these clusters had expressions of *Nfatc1* and *Ctsk*, which are important molecules of osteoclast differentiation [14, 30]. Cluster 5 was detected in Day 3 data (Fig. 4b) and showed the highest expression of osteoclast marker genes (Fig. 4c). These results suggest that cluster 1 contained monocytic progenitors, clusters 0 and 2 contained preosteoclasts, and cluster 5 contained mature osteoclasts (Fig. 4a). Other clusters were annotated with reference to the marker genes from our previous report (Fig. 4a and Supplementary Fig. 3) [31]. Subsequently, we assessed the expression of *Nup85*, *Ccr2*, and *Ccr5* in these clusters and found that all of them were expressed in the monocytic progenitors (cluster 1) (Fig. 4d, e). Given these in vitro results, we used flow cytometry analysis to examine further the expression of CCR2 and CCR5 in OCPs derived from BM cells of ovariectomized mice treated with or without DSF. OCPs were defined as Lin^–^, CD11b^–/lo^, CD115^+^ CD117^+^ [32–34]. The fractions of CCR2- and CCR5- positive cells among the OCPs of ovariectomized mice treated with vehicle were increased, whereas those in the OCPs of ovariectomized mice treated with DSF were either decreased or remained unchanged (Fig. 5a, b). These findings suggest that DSF administration has the potential to affect the expression of DSF target proteins in OCPs.

### DSF inhibited osteoclastogenesis from its early stage

**Fig. 6:**
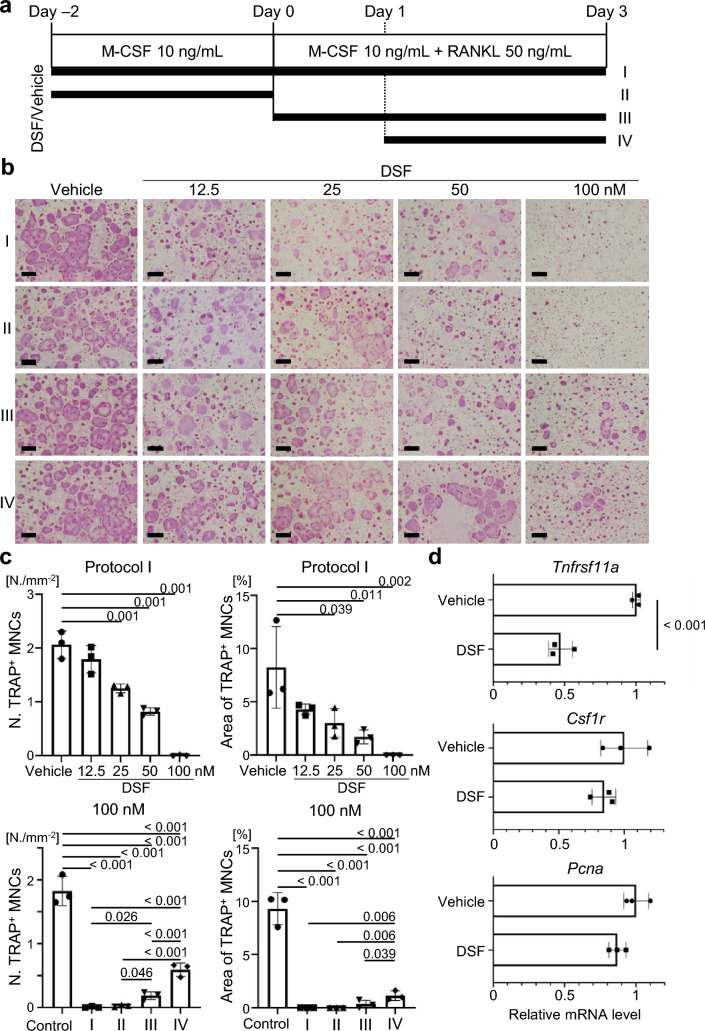
The inhibitory effect of DSF on osteoclastogenesis is observed from an early stage in vitro. **a** Schematic diagram of the time schedule for the exposure of cultured mouse osteoclasts to DSF or vehicle. **b** Representative images of TRAP staining of cultured murine osteoclasts. Scale bars, 100 μm. **c** Numbers of TRAP-positive multinucleated (i.e., more than three nuclei) osteoclasts in areas (N./mm^2^). **d** Relative mRNA expression levels of *Tnfrsf11a*, *Csf1r*, and *Pcna* genes at Day 0. Data are presented as the mean (SD), and *p-*values were determined using Tukey’s multiple comparisons test or Student’s t-test. Significant *p*-values are shown

To further investigate the effect of DSF administration on osteoclast-lineage cells, particularly OCPs, we performed an osteoclastogenesis assay. Murine BM cells were cultured with DSF at various concentrations (12.5 nM, 25 nM, 50 nM, or 100 nM) or vehicle control under different administration conditions according to a previously reported protocol [31]. The DSF administration conditions were designated as follows: Protocol I (DSF was administered throughout the culture period), Protocol II (DSF administered only before the RANKL stimulation), Protocol III (DSF was administered concurrently with RANKL stimulation and continued until the end of the culture period), and Protocol IV (DSF was administered at 24 h after the RANKL stimulation and continued until the end of the culture period) (Fig. 6a). Since vehicle administration in all conditions did not inhibit the differentiation of osteoclasts (Supplementary Fig. 4a), the vehicle data from Protocol I were used as the control in Fig. 6c. In Protocol I, DSF showed a dose-dependent inhibition of the differentiation of TRAP-positive multinucleated osteoclasts (i.e., more than three nuclei) in terms of both the number of cells and the area occupied within the culture dish (Fig. 6b, c and Supplementary Fig. 4b, c). The number of TRAP-positive multinucleated osteoclasts was significantly reduced at a concentration of 25 nM DSF in Protocol I but not in Protocol IV (Supplementary Fig. 4b). This is consistent with the previous report indicating a weak inhibitory effect of DSF on osteoclast differentiation at later stages [23]. Given that OCPs expressed proteins associated with DSF targets (Fig. 4 and Fig. 5), we evaluated the inhibitory effect of DSF on OCPs using Protocol II, where DSF administration predominantly affected OCPs. DSF administration at the OCP stage markedly inhibited osteoclast formation at a concentration of 100 nM (Fig. 6c). This inhibitory effect was comparable to Protocol I (DSF was administered continuously throughout the period). Furthermore, the inhibitory effect on osteoclastogenesis observed in Protocol II was higher than that in Protocols III and IV (Fig. 6c). These data suggest that DSF administration could have a crucial impact on OCPs. To elucidate the action of DSF on the OCPs, we focused on the cells before they received RANKL stimulation, corresponding to “Day 0” in Fig. 6a. qPCR assays revealed that DSF administration suppressed the expression of *Tnfrsf11a*, which is the receptor for RANKL, without affecting *Csf1r* and *Pcna*, a proliferation marker (Fig. 6d). In addition, to evaluate DSF’s potential as a therapeutic agent, we investigated its efficacy following the onset of postmenopausal osteoporosis, which more closely reflects the clinical setting. TNFRSF11A-positive cells in CCR2-positive and CCR5-positive osteoclast precursors were determined using flow cytometry analysis. Our results demonstrated that, even after OVX surgery, TNFRSF11A-positive cells in these cell populations remained comparable to those in sham-operated mice (Supplementary Fig. 5). There is potential for DSF to act on osteoclast precursor cells in postmenopausal conditions. Furthermore, DSF in vivo treatment decreased the number of TNFRSF11A-positive cells in CCR2-positive and CCR5-positive cells in the bone marrow (Supplementary Fig. 6). These findings suggest that DSF prevents osteoclastogenesis by suppressing *Tnfrsf11a* expression at an early stage.

## Discussion

In this report, we demonstrated that DSF, which is used as an anti-alcohol abuse drug, ameliorates the osteoporotic changes and the increase in osteoclast numbers induced by OVX. In addition, DSF did not significantly affect osteoblastic parameters evaluated in OVX model mice or the osteoblast differentiation in in vitro culture. Our previous report showed that DSF suppresses the function of CCR2 through FROUNT, a transcript of *Nup85*. Using scRNA-seq analysis, we found that OCPs express *Ccr2*, *Ccr5*, and *Nup85*. CCR2 or CCR5 deficiency leads to the impairment of osteoclast differentiation [35, 36]. Based on our present results and previous reports, it is predicted that DSF has effects on OCPs through FROUNT, as a common regulator of CCR2 and CCR5 signaling. Interestingly, the fraction of CCR2- and CCR5- positive cells in the OCPs of ovariectomized mice increased, and the increase was mitigated by DSF administration. In other words, DSF has a modulating effect on OCPs concerning the enhanced osteoclast activation seen in OVX model mice. Furthermore, we found that DSF administration had an inhibitory effect on osteoclast differentiation, especially in the early phase, by suppressing the expression of *Tnfrsf11a*, which is a key molecule in osteoclast differentiation. Taken together, we show that DSF has a suppressive effect on OCPs regarding osteoclast differentiation and could be a candidate for osteoporosis treatment without having a significant change on osteoblasts.

There are some reports about the effect of DSF on bone metabolism. As previously mentioned, it has been reported that DSF suppresses osteoclastogenesis through the impairment of RANKL-signaling molecules such as ERK, NF-κB, and NFATc1. Mittal et al. focused on the role of DSF as an ALDH and reported that it suppressed the survival and differentiation of osteoblasts expressing *Aldh2* in their experiments on rats [37, 38]. Regarding the effects of DSF on bone metabolism other than those mentioned above, excluding tumors and bone metastasis, DSF is reported to suppress ethanol-promoted RANKL-induced osteoclastogenesis and osteoporosis in a mouse model [39]. In addition, a previous study by Peris et al. showed that DSF treatment combined with alcohol abstinence (for up to 2 years) increases lumbar and femoral BMD in human alcoholic patients [40]. Given these reports and our results, the dominant effect of DSF administration in ovariectomized mice is mainly the suppression of bone resorption due to the inhibition of osteoclast-lineage cells.

In the osteoclast differentiation cascade, M-CSF acts on the proliferation of OCPs [41, 42]. It has been reported that DSF has an inhibitory effect on the phosphorylation of ERK [23], which is a downstream molecule of M-CSF and RANKL signaling [43]. Therefore, we examined the expression of *Pcna* in the cultured cells at “Day 0” to assess the proliferation potential [44, 45] and found that DSF administration did not affect the expression of *Pcna*. Associated with this result, DSF did not significantly suppress the expression of *Csf1r* but did suppress the expression of *Tnfrsf11a*. These results suggest that DSF affected the OCPs before receiving RANKL stimulation and suppressed the responsiveness of OCPs to RANKL, but not to M-CSF or proliferation.

BM cells from *Ccr2*- or *Ccr5*-deficient mice exhibit impaired osteoclast differentiation [35, 36]. *Ccr2*-deficient mice are known to be protected from OVX-induced bone loss, and this protective effect is mediated via osteoclasts [35]. Furthermore, in an in vitro osteoclastogenesis assay, the stimulation of CCR2 with its ligands, which are C–C motif chemokine ligand (CCL) 2 and CCL7, upregulates Tnfrsf11a expression on preosteoclasts of wild-type mice but not *Ccr2*-deficient mice [35]. These findings support our data, indicating that DSF, by suppressing the CCR2 pathway, ameliorates OVX-induced bone loss and decreases Tnfrsf11a expression in preosteoclasts. Furthermore, osteoclasts derived from *Ccr5*-deficient mice exhibit podosome disarrangements, and the blockade of CCR5 with an anti-hCCR5 antibody demonstrated a suppressive effect on osteoclastogenesis [36]. To summarize these previous reports and our data, the suppressive effect of DSF on in vitro osteoclastogenesis is mediated by FROUNT, CCR2, and CCR5.

Our present results demonstrated the therapeutic potential of DSF for bone metabolic diseases caused by hormone reduction, such as menopause. However, there are other diseases in which bone loss or bone destruction caused by osteoclast activation is also a problem (e.g., inflammatory bone-destructive diseases such as rheumatoid arthritis). This is thought to be due to the remarkable local activation of osteoclasts by pro-inflammatory cytokines. Because DSF acts on osteoclast precursor cells, it may be possible to control their activation before they become mature osteoclasts. Future research is hoped to clarify the effect of DSF on suppressing inflammatory bone destruction. Furthermore, while we demonstrated that DSF affects osteoclast differentiation, considering that DSF also regulates the migration of CCR2- and CCR5-positive cells [16], it is plausible that DSF may influence the migration of osteoclast precursor cells, potentially exerting an inhibitory effect. However, due to the challenges in establishing an experimental system to specifically track migration to bone, this aspect was not investigated in the current study and remains a topic for future research.

There is evidence of increased BMD following oral DSF administration in male alcoholic patients [40]; however, no studies have specifically measured bone density in women after DSF treatment. In addition, we did not use male mice in our experiments. Although potential gender differences in the effects of DSF are not yet clear, our findings suggest that DSF may be a promising candidate for osteoporosis treatment.

In conclusion, DSF attenuates bone loss in ovariectomized mice and suppresses osteoclastogenesis in vivo. The reduction of *Tnfrsf11a* expression in OCPs is a potent candidate for the inhibitory mechanisms of osteoclastogenesis. Osteoblast function is impaired by potent anti-resorptive drugs, but DSF administration does not affect it. Furthermore, the long-term use of DSF for treating chronic alcoholism has demonstrated its safety for the human body. The present data demonstrate the potential of DSF in the treatment of postmenopausal osteoporosis.

## Supplementary Information

Below is the link to the electronic supplementary material.

Supplementary file1 (PDF 1116 KB)

## Data Availability

All data generated or analyzed during this study are included in this published article.
