# Supplementary material for: Disulfiram ameliorates bone loss in ovariectomized mice by suppressing osteoclastogenesis
Source: J Bone Miner Metab. 2024 Oct 7;43(2):61–73. doi: 10.1007/s00774-024-01555-x (PMC11993463; doi:10.1007/s00774-024-01555-x)
Supplement: Supplementary file 1 — Supplementary file1 (PDF 1116 KB) [file 774_2024_1555_MOESM1_ESM.pdf]

Supplementary table and figures

Article title:
"Disulfiram ameliorates bone loss in ovariectomized by suppressing osteoclastogenesis"

Journal name: Journal of Bone Mineral Metabolism

Author names: Tatsuyuki Fukui, Asuka Terashima, Yasunori Omata, Ryota Chijimatsu, Kazuo Okamoto, Masayuki Tsukasaki, Yukiko Fukuda, Tadayoshi Hayata, AkiyoshiSaitoh, Etsuko Toda, Hiroshi Takayanagi, Sakae Tanaka, Yuya Terashima, and Taku Saito

Corresponding author: Asuka Terashima

Bone and Cartilage Regenerative Medicine, Graduate School of Medicine, The University of Tokyo

E-mail: tera-ort@m.u-tokyo.ac.jp

Supplementary Table 1. Primers used for qPCR

CD115 (Csf1r)	F	GACTCTCCAACCTGCATCGG
	R	CCCGGCTCTACAACCAGTTC
Hprt1	F	GGTTAAGCAGTACAGCCCCA
	R	TCCAACACTTCGAGAGGTCC
Pcna	F	GAGAGCTTGGCAATGGGAACA
	R	CAAACGTTAGGTGAACAGGCT
Rank (Tnfrsf11a)	F	CGGAGCTCAGCATCCCTC
	R	CCCCTGGTGTGCTTCTAGC

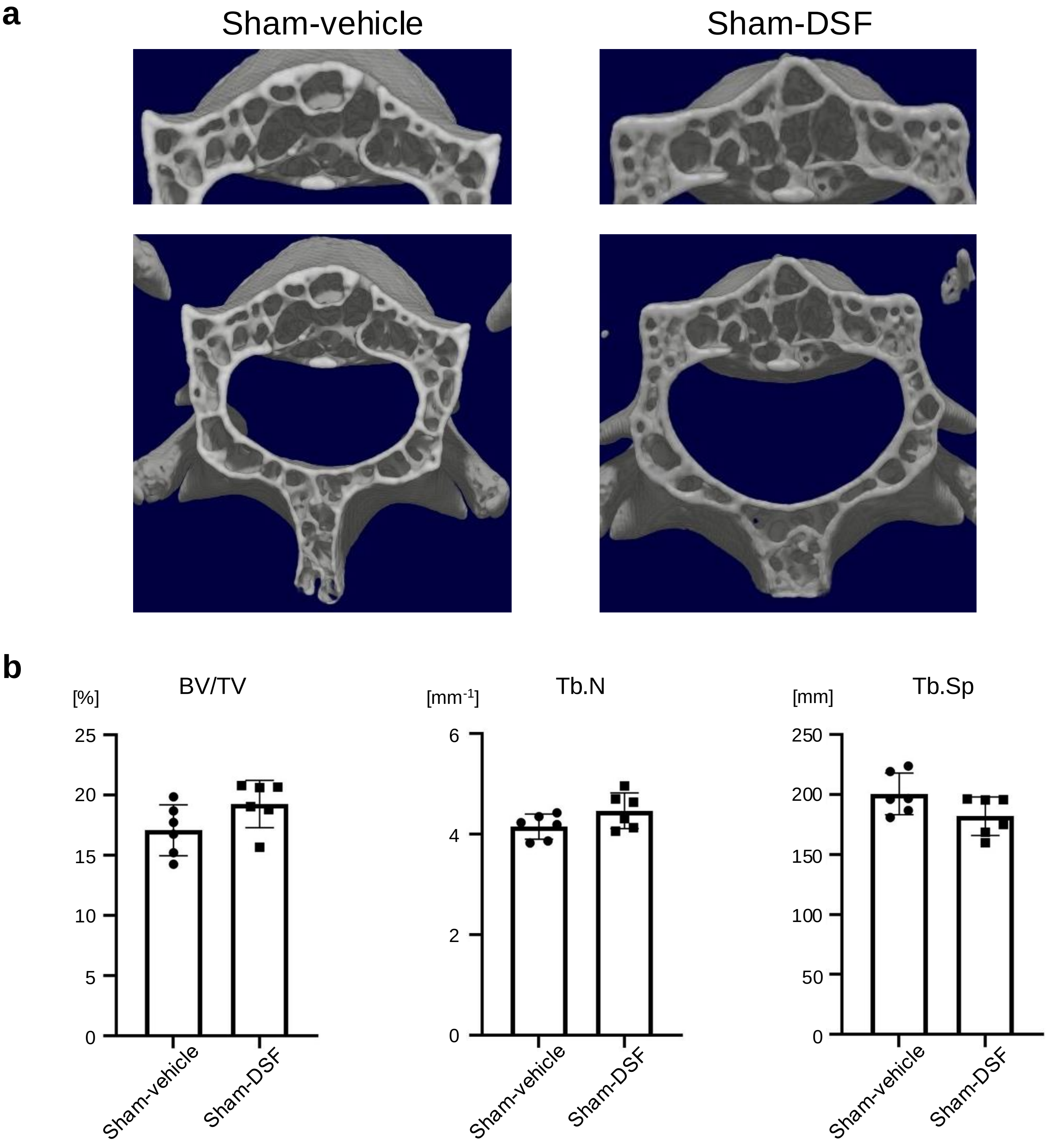

Supplementary Fig. 1. Representative μCT images and vertebral architecture. **a.** Representative images of μCT images of lumbar vertebrae of sham-operated mice treated with vehicle or DSF. **b.** vertebral architectures. Data are presented as the mean (SD), and *p*-values were determined using Student's t-test. Significant *p*-values are shown.

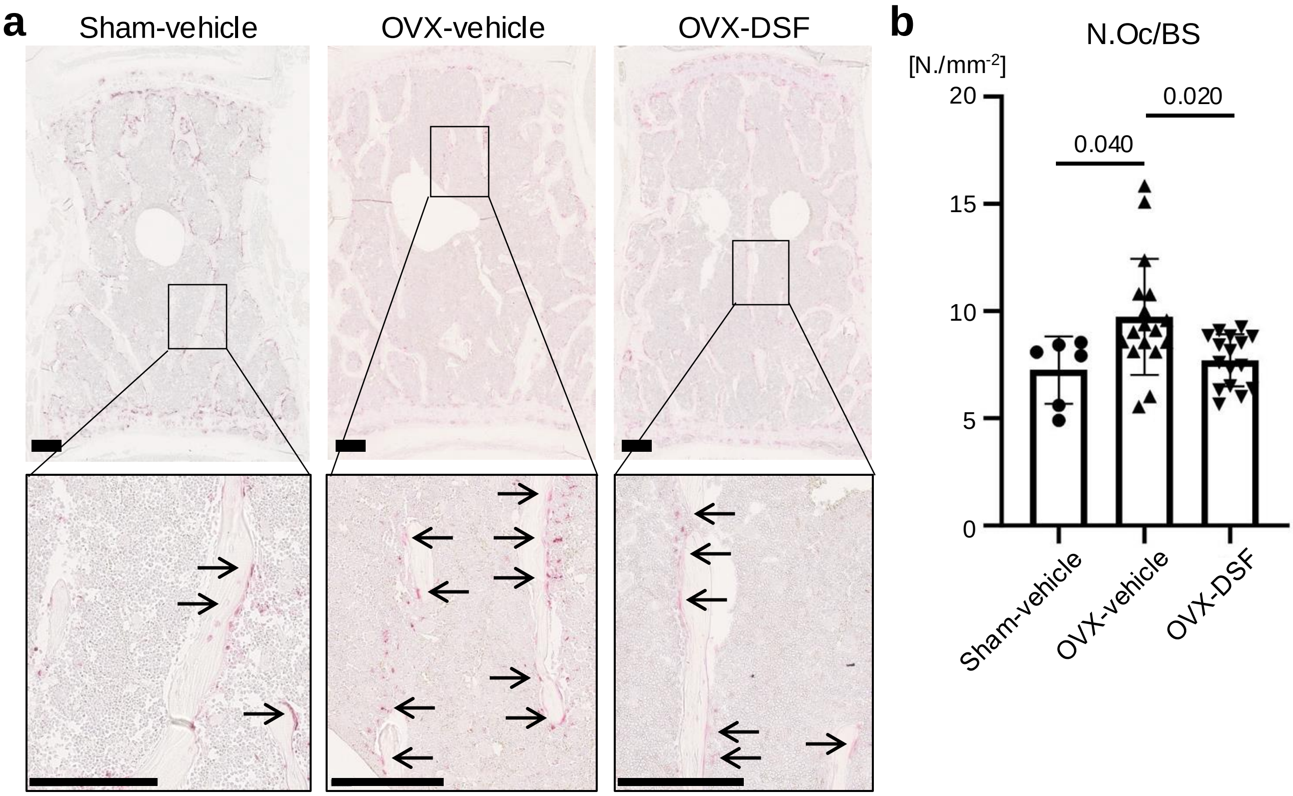

Supplementary Fig. 2. TRAP staining of the vertebral tissue section of eight weeks after OVX. **a**. Representative images of TRAP staining of the vertebral tissue sections. Arrows indicate osteoclasts. Scale bars, 100 μm. **b**. Quantitative analysis of TRAP-positive osteoclast numbers. Data are presented as the mean (SD). *p* values were measured using Tukey's multiple comparisons test. Significant *p* values are shown.

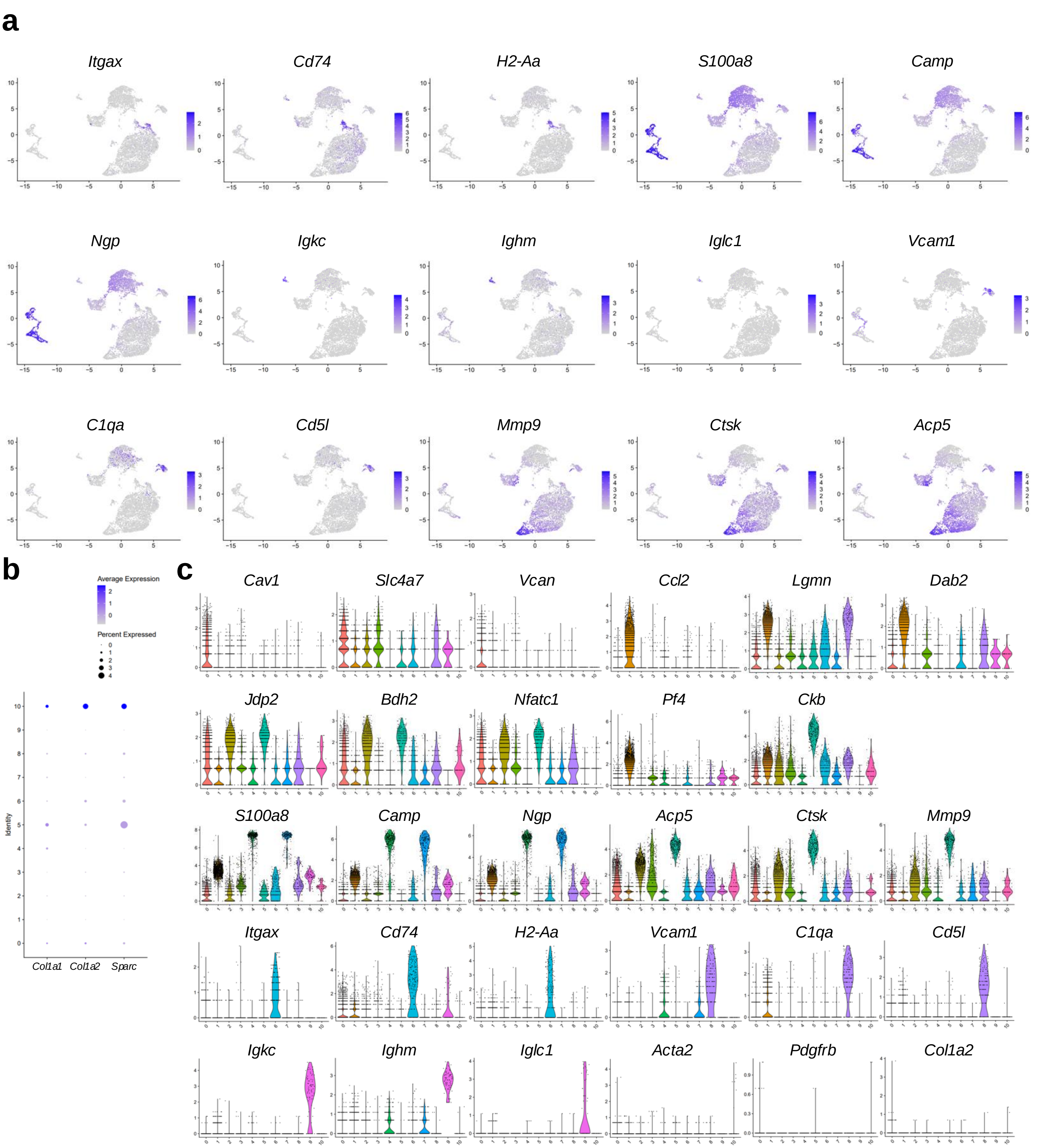

Supplementary Fig. 3. The characteristics of each cluster. **a**. The expressions of *Itgax, Cd74, H2-Aa* (for DC-like precursors), *S100a8, Camp, Ngp* (for neutrophils), *Igkc, Ighm, Iglc1* (for B cells), *Vcam1, C1qa, Cd5l* (for Vcam1^{+} macrophage), *Mt3, Mmp9, Ctsk, and Acp5* (for osteoclasts and Mt3^{hi} cells) in the UMAP visualization. **b**. Dot plots showing the expressions of *Col1a1, Col1a2, and Sparc* (for mesenchymal stromal cells) in the identified clusters. **c**. Violin plots of marker genes for various cell types in the identified clusters. The marker genes were selected with reference to the previous report [31].

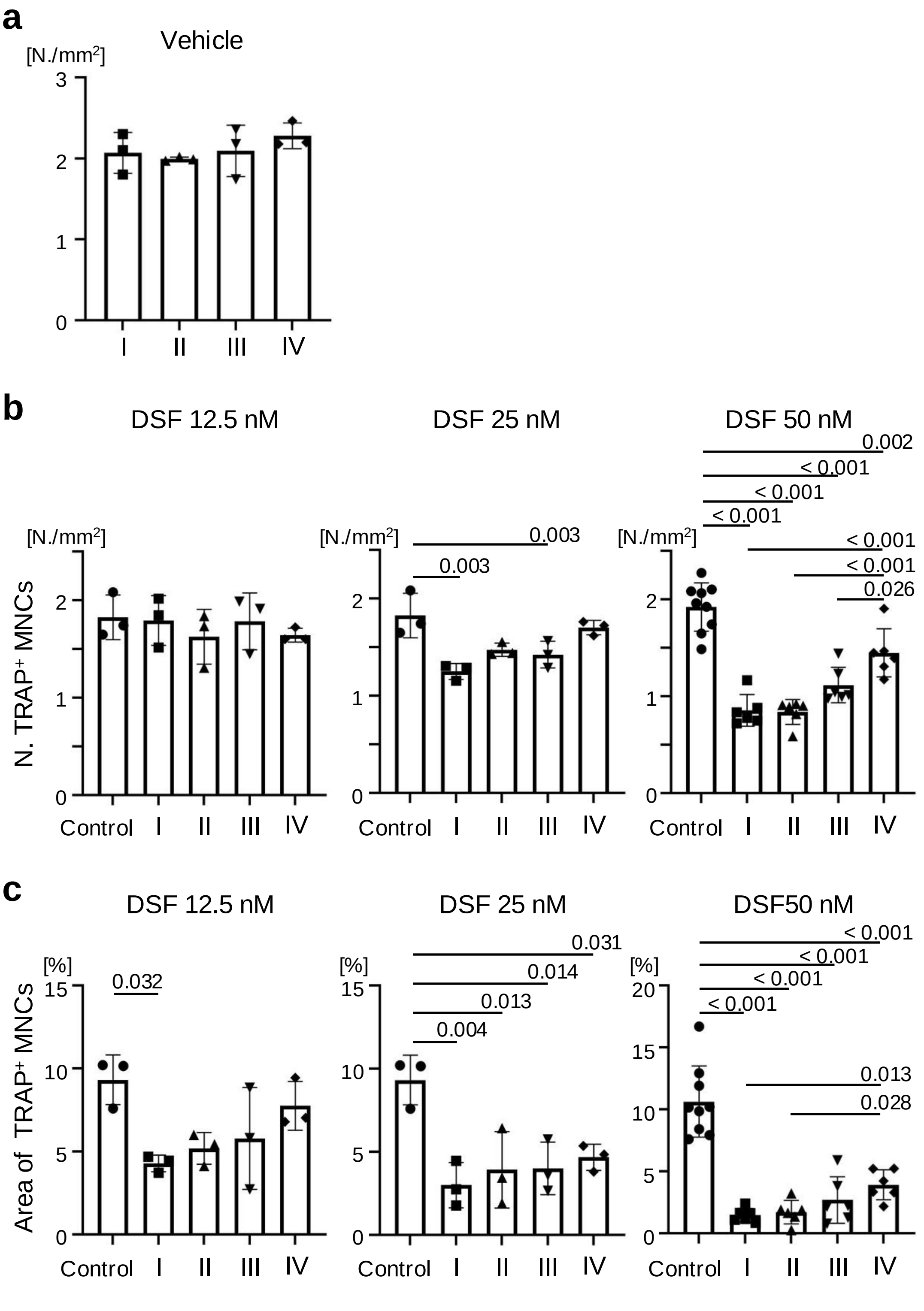

Supplementary Fig. 4. Osteoclast differentiation at various DSF concentrations and administration conditions. **a** The numbers of TRAP-positive multinucleated osteoclasts treated with various concentrations of vehicle. The numbers (**b**) and areas (**c**) of TRAP-positive multinucleated osteoclasts. Data are presented as the mean (SD). *p* values were measured using Tukey's multiple comparisons test. Significant *p* values are shown.

a

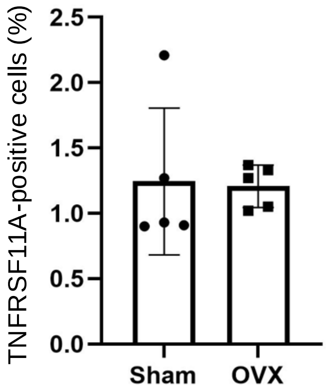

b

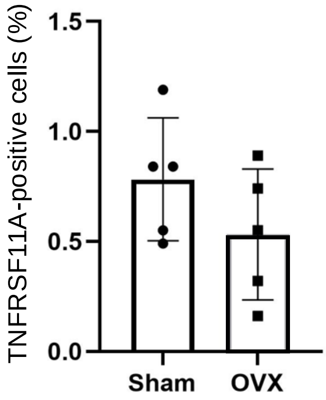

Supplementary Fig. 5. TNFRSF11a positive cells in OCPs after OVX treatment. Percentage of TNFRSF11A positive cells in CCR2- (a) or CCR5- positive cells (b) in Lin^{-} $CD11b^{-/lo}$ $CD115^{+}$ $CD117^{+}$ OCPs. Data are presented as the mean (SD), and *p*-values were determined using Student's t-test. Significant *p*-values are shown.

a

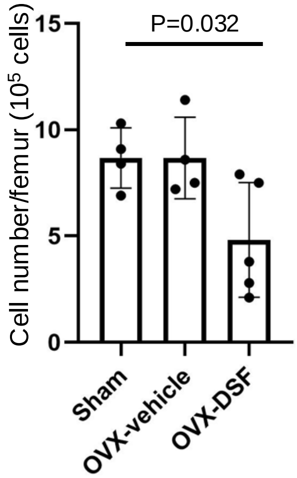

b

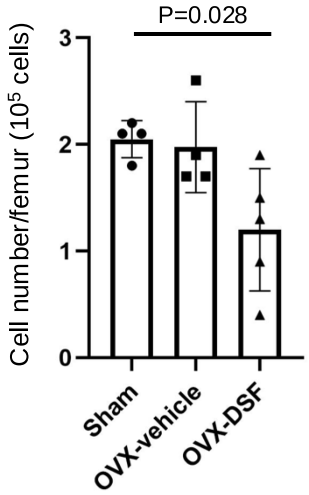

Supplementary Fig. 6. TNFRSF11A-positive cells. The number of TNFRSF11A-positive cells in CCR2- (a) or CCR5- positive cells (b) in bone marrow. *p* values were measured using Tukey's multiple comparisons test. Significant *p* values are shown.
